# Tumors and Their Microenvironment Dual‐Targeting Chemotherapy with Local Immune Adjuvant Therapy for Effective Antitumor Immunity against Breast Cancer

**DOI:** 10.1002/advs.201801868

**Published:** 2019-01-30

**Authors:** Caifeng Deng, Quan Zhang, Mengdi Jia, Jin Zhao, Xun Sun, Tao Gong, Zhirong Zhang

**Affiliations:** ^1^ Key Laboratory of Drug‐Targeting and Drug Delivery System of the Education Ministry Sichuan Engineering Laboratory for Plant‐Sourced Drug and Sichuan Research Center for Drug Precision Industrial Technology West China School of Pharmacy Sichuan University Chengdu 610064 P. R. China; ^2^ School of Pharmacy Chengdu Medical College Chengdu 610083 China

**Keywords:** antitumor immunity, chemotherapy‐induced “vaccines”, effector T cells activation, M2‐tumor associated macrophages, tumor immunosuppression

## Abstract

Chemotherapy turns tumor cells into “tumor vaccines” by immunogenic cell death (ICD). However, it remains a challenge to exploit chemotherapy‐induced “tumor vaccines” for solid cancer immunotherapy due to the inefficient effector T cells activation and tumor microenvironment immunosuppression. Here, a matrix metalloprotease 2 responsive liposome (PEG‐FA‐Lip) composed of cleavable PEG chains covering the folate (FA)‐modified liposome is developed to deliver ICD inducer doxorubicin. In breast cancer‐bearing mice, PEG‐FA‐Lip targets both 4T1 breast cancer cells and M2‐tumor associated macrophages (M2‐TAMs) via FA‐receptor mediated endocytosis, resulting in abundant “tumor vaccines” and efficient elimination of M2‐TAMs. The combination of local cytosine‐phosphate‐guanine (CpG) therapy facilitates PEG‐FA‐Lip induced “tumor vaccines” to effectively arouse systematic effector T cells immune response through promoting dendritic cell maturation and immunostimulatory cytokines secretion. The simultaneous elimination of M2‐TAMs ensures the activated effector T cells exert antitumor immunity within tumor via decreasing immunosuppressive cytokines secretion and tumor infiltration of Treg cells. After receiving the combined treatment, 30.1% of breast cancer‐bearing mice (initial tumor volume > 100 mm^3^) achieves the goal of tumor eradication. Remarkably, this combination therapy greatly inhibits lung metastasis and controls the growth of already metastasized breast cancers (initial tumor volume > 100 mm^3^).

## 1 Introduction

Immunotherapy has shown tremendous promises as a next generation treatment strategy for cancer therapy.^1^ Among the developed immunotherapy strategies, tumor vaccines could be an ideal way to eradicate cancers and prevent tumor metastasis by inducing antigen‐specific effector T cells against tumors, rather than nonspecific immunological responses triggered by cytokine therapy or checkpoint‐blockade therapy.[[qv: 1a,2]] Although conventional cancer vaccines could induce tumor antigen‐specific effector T cells, their clinical applications were not satisfying mainly due to the tumor heterogeneity of patients and complicated manufacture process.^3^ Furthermore, the antitumor efficacy of immunotherapy‐induced effector T cells could be discounted on solid tumors.^4^ Therefore, it is urgent needed to develop a cancer immunotherapy strategy that could effectively generate highly immunogenic tumor vaccines to trigger robust effector T cells immune response and simultaneously ensure the antitumor efficacy of effector T cells on solid tumors.

Chemotherapeutic drugs have played an important role in treating cancers because of their wide antitumor spectrum and direct cytotoxicity to tumor cells.^5^ Interestingly, recent advances have demonstrated that some chemotherapeutics including doxorubicin (DOX), cyclophosphamide, and cisplatin could act as immunogenic cell death (ICD) inducers by arousing calreticulin (CRT) exposure on tumor cell surface and the release of high mobility group box 1 (HMGB1) from tumor cells.^6^ CRT exposure and HMGB1 release induced by those chemotherapeutics could increase the immunogenicity of tumor cells and facilitate dying tumor cell phagocytosis by dendritic cells (DCs).^7^ In addition, tumor‐associated antigens could be released from chemotherapy induced dying tumor cells when ICD occurs. Those dying tumor cells could work as whole‐cell cancer vaccines, which can induce immunities against all released potential tumor antigens.^5^ Therefore, chemotherapy could produce “tumor vaccines” in tumor sites and overcome tumor heterogeneity.^7^ Taken these into consideration, chemotherapy might represent a promising and convenient way to gain highly immunogenic tumor vaccines for cancer immunotherapy.

Despite potential, the antitumor immunity triggered by chemotherapeutics induced vaccines was limited,[[qv: 6a,8]] which was mainly on account of inefficient effector T cells activation and immunosuppression in tumor microenvironment. Chemotherapeutic drug with low molecular weight were tend to distribute widely throughout the body, resulting in less than 1% of the intravenously injected chemotherapeutics could distribute in tumors.^9^ With limited drug distribution in tumors, chemotherapy could not effectively produce “tumor vaccines.” Besides, the efficient induction of effector T cells depends upon tumor vaccines reaching and being presented to naive T cells in lymph nodes.^10^ Immature DCs transport tumor vaccines from tumor sites to local lymph nodes, and mature DCs are of critical importance to antigen presentation.^11^ Tumor‐draining lymph nodes (TDLNs) are in close proximity to tumor sites and most immature DCs from tumor sites would migrate to TDLNs after capturing tumor antigens.^12^ However, the DC maturation and activation within the TDLNs are always inhibited,^13^ leading to less effective activation of naive T cells into effector T cells even in the presence of highly immunogenic tumor vaccines.^14^ Accordingly, with limited induction of “tumor vaccines” in tumor sites and inhibited DC maturation in TDLNs, chemotherapeutics often fail to efficiently induce effector T cells activation. Furthermore, an increasing number of studies have shown that the immunosuppressive tumor microenvironment of solid tumors could inhibit effector T cells attacking tumor cells.^15^ In particular, M2‐tumor associated macrophages (M2‐TAMs), as critical modulators of the tumor microenvironment, have played a vital role in antitumor immunity suppression.^16^ On the one hand, M2‐TAMs can secrete large amounts of immunosuppressive cytokine including TGF‐β and IL‐10 to limit the activity of effector T cells and terminate antitumor immunity.^17^ On the other hand, M2‐TAMs also facilitate tumor infiltration of regulatory T cells (Treg cells). Studies proved that Treg cell as a prominent immunosuppressive immune cell could disable effector T cells, thus inhibiting effector T cells attacking tumor cells.^17^, ^18^ Therefore, effectively facilitating chemotherapy‐induced “tumor vaccines” to induce effector T cells activation and simultaneously overcoming the M2‐TAMs mediated tumor microenvironment immunosuppression is a promising strategy for solid tumor immunotherapy.

**Figure 1:**
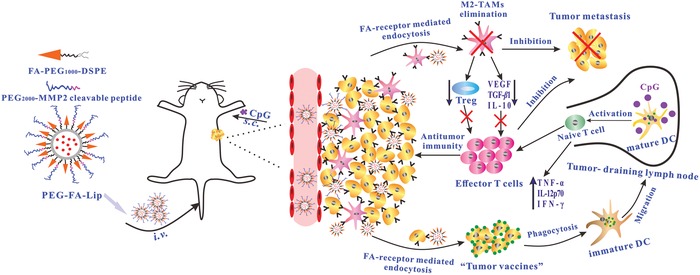
The mechanism of antitumor immunity triggered by PEG‐FA‐Lip based targeting chemotherapy in combination with CpG immune adjuvant therapy. Abbreviations: s.c., subcutaneous injection; i.v., intravenous injection.

In this study, the combination of a matrix metalloprotease 2 (MMP2) responsive folate (FA)‐modified liposome (PEG‐FA‐Lip) with local immune adjuvant therapy was adopted to treat solid tumor‐breast cancer. With long PEG chains modification, DOX‐loaded PEG‐FA‐Lip was expected to exhibit extended blood circulation and increased tumor distribution through enhanced permeation and retention (EPR) effect.^19^ When reaching tumor sites, PEG‐FA‐Lip could have the detachment of long PEG chains after responding to MMP2 in tumor microenvironment.^20^ Consequently, FA was exposed and DOX was targetedly delivered to both tumor cells and M2‐TAMs through FA‐receptor mediated endocytosis rather than immunocytes as FA receptor is overexpressed on breast cancer cells^21^ and M2‐TAMs.^22^ The FA‐mediated tumor cells targeting would induce abundant “tumor vaccines” through arousing increased ICD. Immature DCs in tumor sites capture PEG‐FA‐Lip induced “tumor vaccines” and subsequently migrate into TDLNs. Then the combined immune adjuvant cytosine‐phosphate‐guanine (CpG) could facilitate PEG‐FA‐Lip induced “tumor vaccines” effectively trigger effector T cells activation in TDLNs by promoting DC maturation and increasing immunostimulatory cytokines secretion. Finally, the FA‐mediated M2‐TAMs targeting would remold the immunosuppressive tumor microenvironment, which could ensure activated effector T cells home to tumor sites and play antitumor immunity in tumors. Taken together, the combination of PEG‐FA‐Lip with CpG caused regression and metastatic inhibition of 4T1 breast cancers by efficiently inducing effector T cells activation and reversing the M2‐TAMs mediated immunosuppression in tumor microenvironment (**Figure** **1**).

## 2 Results and Discussion

### 2.1 Characterization and Dual‐Targeting Efficacy of PEG‐FA‐Lip

**Figure 2:**
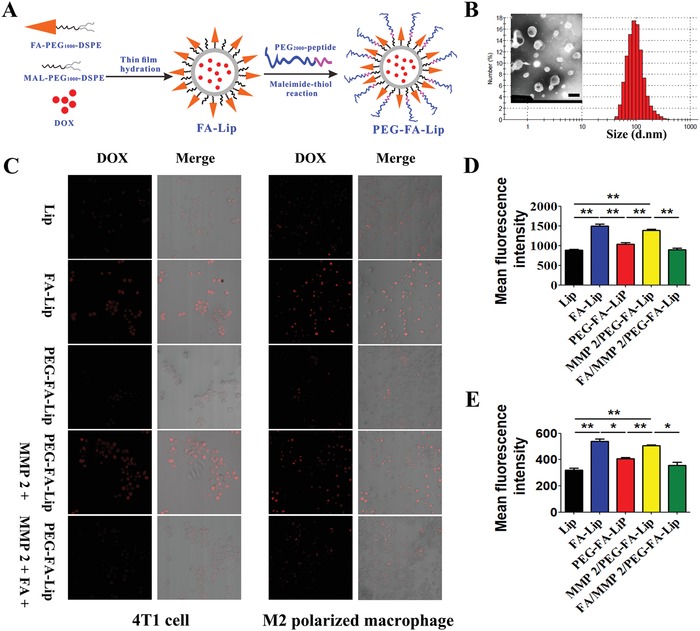
In vitro characterization of PEG‐FA‐Lip and evaluation of its dual‐targeting efficacy. A) Schematic illustration of preparing PEG‐FA‐Lip. B) Size distribution and TEM image of PEG‐FA‐Lip. Scale bar = 200 nm. C) Confocal images (200×) of cellular uptake on 4T1 cells and M2 polarized macrophages (RAW 264.7 cells with IL‐4). D,E) Quantitative cellular uptake of Lip, FA‐Lip, and PEG‐FA‐Lip on 4T1 cells (D) and M2 polarized macrophages (E) after incubation for 1 h at the DOX concentration of 10 µg mL^−1^. * *P* < 0.05, ** *P* < 0.01. Data represent the mean ± SD (*n* = 3).

Nanocarriers such as biomolecular delivery platforms^23^ and lipid‐based drug delivery systems^24^ are widely used to selectively deliver antitumor drugs into tumors. Among them, liposomes show great biocompatibility and have been successfully applied in clinic. Therefore, liposomes were adopted as drug carrier in this work. We prepared liposomes by the thin‐film hydration method^25^ and PEG_2000_‐MMP2 cleavable peptide was linked to FA‐modified liposomes (FA‐Lip) through maleimide‐thiol reaction (**Figure** **2**A). The particle size of FA‐Lip was 127.2 ± 4.9 nm. After being covered by PEG_2000_ blocks, the particle size of PEG‐FA‐Lip slightly increased to 138.5 ± 6.8 nm. The average zeta potential of FA‐Lip and PEG‐FA‐Lip were −6.7 ± 0.6 mV and −9.3 ± 0.8 mV, respectively. The DOX encapsulation efficiencies of various prepared liposomes were all greater than 90% (Table S1, Supporting Information). The transmission electron microscope (TEM) images revealed that the morphologies of PEG‐FA‐Lip were generally spherical (Figure 2B).

To confirm that PEG‐FA‐Lip could dual‐target tumor cells and M2‐TAMs, the cellular uptake study was conducted. Confocal images showed that FA‐Lip significantly increased the red fluorescent intensity of DOX compared with common liposomes (Lip) in both 4T1 tumor cells and M2 polarized macrophages (Figure 2C). Such observations were not seen in M1 polarized macrophages (Figure S2, Supporting Information). These results implied that the FA‐Lip could target both tumor cells and M2‐TAMs. In addition, PEG‐FA‐Lip remarkably decreased the intensity of red fluorescence in all treated cells. However, with the presence of MMP2, the fluorescent intensity decrease of PEG‐FA‐Lip was reversed in 4T1 tumor cells and M2 polarized macrophages, suggesting that PEG‐FA‐Lip could respond to MMP2 and then target both tumor cells and M2‐TAMs. To further evaluate the FA mediated‐endocytosis, the FA competitive inhibition assay was performed. We observed that preincubation of FA significantly decreased the fluorescence of PEG‐FA‐Lip (in the presence of MMP2) in 4T1 tumor cells and M2 polarized macrophages, whereas in M1 polarized macrophages, the aforementioned differences were not observed (Figure 2C; Figure S2, Supporting Information). The results of quantitative analysis by flow cytometry also showed the same trend (Figure 2D,E). In addition, the results of MTT study indicated that PEG‐FA‐Lip (in the presence of MMP2) had higher cytotoxicity to M2 polarized macrophages than to M1 polarized macrophages (Figure S3, Supporting Information).

We also investigated whether PEG‐FA‐Lip could dually target tumor cells and M2‐TAMs when intravenously injected into 4T1 tumor‐bearing BALB/c mice. It was observed that PEG‐FA‐Lip significantly increased the fluorescence distribution in tumors compared with Lip and FA‐Lip, and reached the maximum at 8 h (Figure S4, Supporting Information). The ex vivo imaging analysis 24 h postinjection and pharmacokinetics evaluation also demonstrated the highest tumor accumulation and the longest blood circulation time of PEG‐FA‐Lip (Figures S4–S7, Supporting Information). Of note, PEG‐FA‐Lip remarkably reduced the fluorescence distribution in liver and spleen compared with FA‐Lip (Figure S4B,C, Supporting Information), which indicated that the nonspecific distribution of FA‐Lip could be avoided by using long PEG chains to cover FA, as FA receptors are also highly expressed in normal tissues including liver and spleen.^26^ In addition, we employed antibodies of F4/80 and CD206 to characterize M2‐TAMs.^27^ Remarkably, the distribution of FA‐Lip and PEG‐FA‐Lip overlapped with the fluorescence of F4/80 and CD206 (Figure S5, Supporting Information), demonstrating the FA‐mediated endocytosis of liposomes on M2‐TAMs. The results also revealed that PEG‐FA‐Lip was tumor microenvironment‐responsive and could target both tumor cells and M2‐TAMs in vivo.

### 2.2 PEG‐FA‐Lip Inducing “Tumor Vaccines” via ICD In Vitro and In Vivo

**Figure 3:**
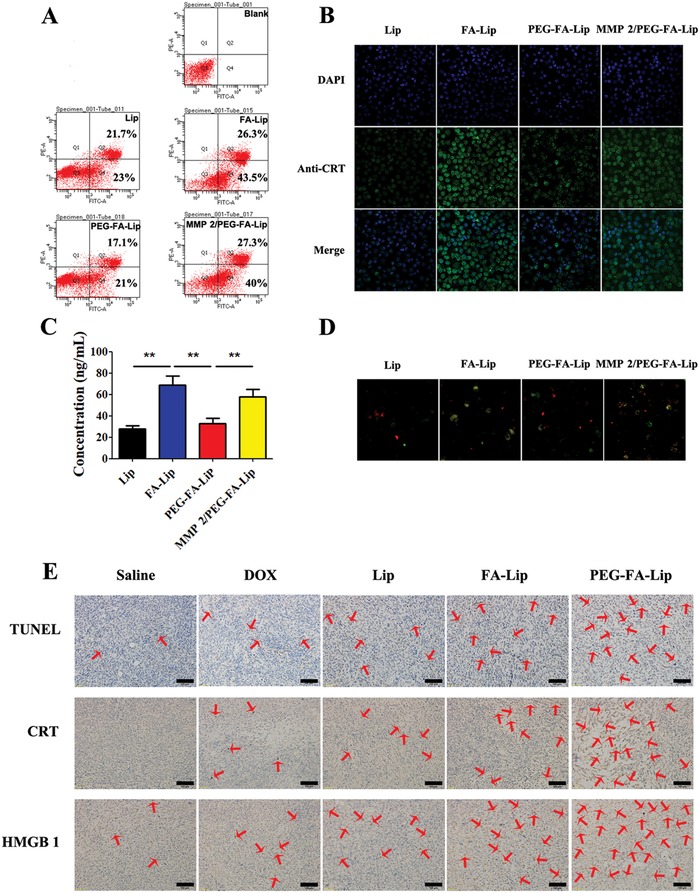
PEG‐FA‐Lip inducing “tumor vaccines” via ICD in vitro and in vivo. A) Flow cytometric analysis of 4T1 cell apoptosis induced by Lip, FA‐Lip and PEG‐FA‐Lip with or without presence of MMP2 for 24 h at the DOX concentration of 400 ng mL^−1^. B) Confocal images (200×) of CRT exposure on the cell surface of 4T1 cells after treated with Lip, FA‐Lip, and PEG‐FA‐Lip with or without presence of MMP2 for 24 h at the DOX concentration of 400 ng mL^−1^. Cell nuclei were stained with DAPI (blue) and CRT (green) were detected by Alexa Fluor 488‐conjugated anti‐CRT antibody staining. C) The concentration of HMGB1 in culture supernatants after 4T1 cells treated with different formulations for 24 h was analyzed by ELISA assay. ** *P* < 0.01. Data represent the mean ± SD (*n* = 3). D) Confocal images (200×) of the DC phagocytizing different liposomes induced apoptotic 4T1 cells. DiO (green fluorescence) stained 4T1 cells were treated with different formulations for 24 h at the DOX concentration of 400 ng mL^−1^ before coincubating with DiI (red fluorescence) stained DC 2.4 cells. E) TUNEL, CRT, and HMGB1 staining of the 4T1 tumor tissues from different groups of breast cancer‐bearing mice after receiving the indicated treatment (*n* = 3). Scale bar = 100 µm.

With tumor cell targeting ability and improved tumor distribution, PEG‐FA‐Lip was expected to be advantageous in efficiently inducing “tumor vaccines” via ICD. ICD occurs when apoptotic tumor cells elicit specific molecular events including CRT exposure and HMGB1 release.^5^, ^28^ The apoptosis of 4T1 tumor cells in this study was determined by flow cytometry assay.^29^ It was shown in **Figure** **3**A that about 70% of 4T1 cells were induced to apoptosis after FA‐Lip treatment, which was much higher than that of Lip and PEG‐FA‐Lip treatment. Furthermore, with the presence of MMP2, PEG‐FA‐Lip also caused about 67% apoptosis of 4T1 cells, demonstrating that PEG‐FA‐Lip was MMP2‐responsive. The translocation of CRT from endoplasmic reticulum to the tumor cell surface was demonstrated by Alexa Fluor 488‐CRT antibody staining.[[qv: 7a]] The confocal images showed that FA‐Lip and PEG‐FA‐Lip with MMP2 caused a higher level of CRT exposure on the 4T1 tumor cell surface (Figure 3B). The levels of HMGB1 in the supernatants of different liposomes treated 4T1 tumor cells were confirmed by enzyme‐linked immunosorbent assay (ELISA).[[qv: 7a]] Compared with Lip, FA‐Lip and PEG‐FA‐Lip with MMP2 increased HMGB1 release from 27.8 to 68.7 and 58.0 ng mL^−1^, respectively (Figure 3C). Those results suggested that both FA‐Lip and PEG‐FA‐Lip in the presence of MMP2 could induce ICD in vitro. In addition, the results of in vivo study showed that PEG‐FA‐Lip caused highest levels of tumor cell apoptosis, CRT exposure, and HMGB1 release among all the treated groups (Figure 3E), demonstrated that PEG‐FA‐Lip could effectively induce ICD in tumor sites.

Chemotherapy induced ICD turns tumor cells into “tumor vaccines” by promoting tumor cells being recognized and processed by DCs.^7^ CRT acts as an “eat me” signal could facilitate the phagocytosis of apoptotic tumor cells by DCs.[[qv: 7a]] HMGB1 acts as a “find me” signal to regulate DC‐mediated tumor antigen cross‐presentation and T‐cell polarization.[[qv: 7a]] To demonstrated PEG‐FA‐Lip treated tumor cells could be effectively captured by DCs, we challenged DCs with various liposomes induced apoptotic 4T1 tumor cells and observed their responses by confocal images. As shown in Figure 3D, FA‐Lip and PEG‐FA‐Lip with MMP2 treated tumor cells displayed a greater extent of phagocytosis by DCs compared with Lip. This observation clarified that FA‐mediated endocytosis increased immunogenicity of DOX treated dying tumor cells and could turn 4T1 tumor cells into “tumor vaccines” via ICD. The results also implied that PEG‐FA‐Lip with the highest level of ICD induction in vivo (Figure 3E) would arousing abundant “tumor vaccines” in tumor sites.

### 2.3 CpG Facilitating “Tumor Vaccines” to Activate Effector T Cells in TDLNs

**Figure 4:**
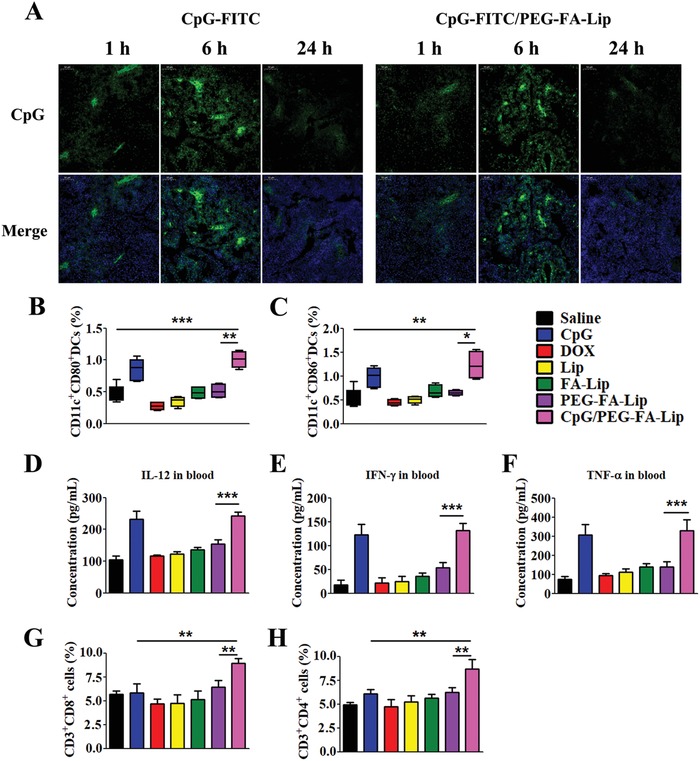
CpG facilitating PEG‐FA‐Lip induced “tumor vaccines” to activate effector T cells in TDLNs. A) Confocal images (200×) showing the distribution of CpG in TDLNs at 1, 6, and 24 h after breast cancer‐bearing mice treated with CpG or CpG/PEG‐FA‐Lip. (CpG was labeled with FITC and is represented in green, nuclei were labeled with DAPI and are represented in blue.) The CpG/PEG‐FA‐Lip group was pretreated with PEG‐FA‐Lip via intravenous injection. The CpG‐FITC was subcutaneously injected into the left axilla of mice at 24 h after PEG‐FA‐Lip treatment. B,C) DC maturation. Proportions of DCs expressing CD80 (B) and CD86 (C) in TDLNs after the indicated treatment. D–F) Immunostimulatory cytokines secretion. ELISA results of IL‐12p70 (D), IFN‐γ (E), and TNF‐α (F) secretion in blood after the indicated treatment. G,H) Effector T cells activation. Relative abundance of CD3^+^CD8^+^ cells (G) and CD3^+^CD4^+^ cells (H) in TDLNs after the indicated treatment. * *P* < 0.05, ** *P* < 0.01, *** *P* < 0.001. Data represent mean ± SD (*n* = 5).

We have proved that PEG‐FA‐Lip could effectively induce “tumor vaccines” within tumors. Then immature DCs would capture “tumor vaccines” and migrate to TDLNs.[[qv: 3b,12b]] The successful induction of effector T cells requires immature DCs activation into mature DCs, and thus, providing the additional costimulatory signals (CD80 and CD86 molecules) for T cell activation.^11^ However, the DC maturation in TDLNs was always inhibited, leading to inefficient T cells activation even in the presence of highly immunogenic vaccines.^13^ To address that problem, the immune adjuvant CpG which could promote DC maturation and immunostimulatory cytokines secretion was always combined with conventional vaccines to increase effector T cells activation.^30^ We wondered whether CpG could also facilitate PEG‐FA‐Lip induced “tumor vaccines” efficiently activate effector T cells. To ensure CpG could reach in TDLNs to play its function, the CpG was subcutaneously injected into the left axilla of breast cancer‐bearing mice. We found that the locally injected CpG could efficiently accumulated in TDLNs within 24 h (**Figure** **4**A). The PEG‐FA‐Lip did not show obvious effect on CpG distribution in TDLNs (Figure 4A). Then we investigated the immunological effects of CpG combining with PEG‐FA‐Lip (CpG/PEG‐FA‐Lip) toward TDLNs‐derived DCs separated from 4T1 tumor‐bearing mice by using flow cytometry. As shown in Figure 4B,C, both CpG and CpG/PEG‐FA‐Lip significantly increased the expression levels of CD80 and CD86 molecules on CD11c^+^ marked DCs compared with saline group, which demonstrated that CpG could efficiently promotes DC maturation in TDLNs.^31^ Upon maturation, DCs would increase the secretion of immunostimulatory cytokines including TNF‐α, IL‐12p70, and IFN‐γ.^31^ The ELISA results revealed that both CpG and CpG/PEG‐FA‐Lip showed much higher levels of those cytokines in sera compared with other groups (Figure 4D–F). These results demonstrated that CpG could promote DC maturation and increase immunostimulatory cytokines. In addition, PEG‐FA‐Lip had no obvious effects on CpG promoting DC maturation and immunostimulatory cytokines secretion.

DC maturation and increased secretion of immunostimulatory cytokines could be advantageous to T cell activation.^32^ To confirm CpG together with PEG‐FA‐Lip could effectively inducing effector T cell activation, we investigated the abundance of effector T cells including CD4+ T cells (CD3^+^CD4^+^ cells) and CD8+ T cells (CD3^+^CD8^+^ cells) in TDLNs. Although CpG significantly promoted DC maturation and increased immunostimulatory cytokines, we found that CpG slightly increased the ratio of effector T cells in TDLNs (Figure 4G,H). The main reason for CpG inducing inefficient effector T cell activation was lacking of efficient exposed tumor antigens. PEG‐FA‐Lip induced abundant “tumor vaccines,” however, results revealed that PEG‐FA‐Lip showed limited effect on increasing the amounts of effector T cells in TDLNs compared with CpG/PEG‐FA‐Lip (Figure 4G,H). That's because the inefficient DC maturation in PEG‐FA‐Lip treated group was greatly inhibited the effector T cells activation. Remarkably, CpG/PEG‐FA‐Lip displayed the highest proportions of CD4+ T cells and CD8+ T cells among all the treated groups (Figure 4G,H), which demonstrated CpG could ensure PEG‐FA‐Lip induced “tumor vaccines” effectively activate effector T cells.

### 2.4 Reprogrammed Immunosuppressive Tumor Microenvironment

**Figure 5:**
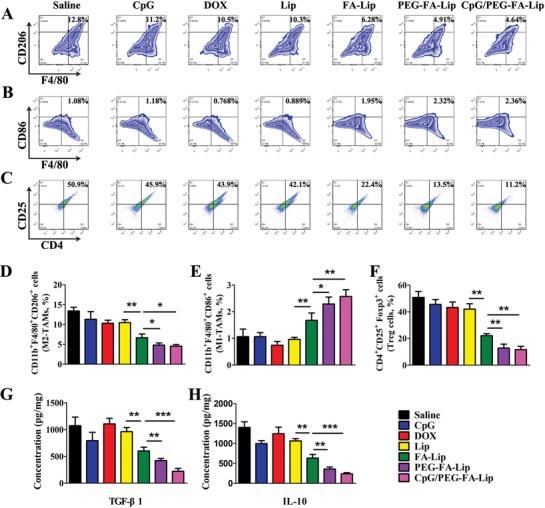
PEG‐FA‐Lip together with CpG reprogramming immunosuppressive tumor microenvironment by eliminating M2‐TAMs. A) Representative flow cytometry profiles of M2‐TAMs (CD11b^+^F4/80^+^CD206^+^ cells) in 4T1 tumors after the indicated treatment. (gated by CD11b^+^ cells). B) Representative flow cytometry profiles of M1‐TAMs (CD11b^+^F4/80^+^CD86^+^ cells) in tumors after the indicated treatment. (gated by CD11b^+^ cells). C) Flow cytometry data showing the fraction of Treg cells (CD4^+^CD25^+^Foxp3^+^ cells) in tumors after the indicated treatment. (gated by Foxp3^+^ cells). D–F) Relative abundance of M2‐TAMs (D), M1‐TAMs (E), and Treg cells (F) in 4T1 tumor tissues after indicated treatment. G,H) ELISA results of TGF‐β1 (G) and IL‐10 (H) secretion in 4T1 tumor tissues from mice receiving the indicated treatment. * *P* < 0.05, ** *P* < 0.01, *** *P* < 0.001. Data represent mean ± SD (*n* = 5).

The combination of PEG‐FA‐Lip with CpG could efficiently induce effector T cells activation in TDLNs. Then the activated effector T cells would migrate into tumor sites to play antitumor immunity. However, the M2‐TAMs mediated immunosuppression in tumor microenvironment could greatly inhibit effector T cells playing antitumor immunity within tumor.^17^, ^18^ Fortunately, PEG‐FA‐Lip was able to target M2‐TAMs via FA receptor endocytosis, which might remold the immunosuppressive tumor microenvironment by selective elimination of M2‐TAMs. To confirm the combination of CpG with PEG‐FA‐Lip could also reduce tumor immunosuppression via eliminating M2‐TAMs, female BALB/c mice bearing 4T1 breast cancers were randomly divided into seven groups including saline, CpG, DOX, Lip, FA‐Lip, PEG‐FA‐Lip, and CpG/PEG‐FA‐Lip. At day 10 and 13, saline and 5 mg kg^−1^ DOX equivalents of DOX, Lip, FA‐Lip, and PEG‐FA‐Lip were intravenously injected into mice. At day 11, 13, 15, and 17, CpG (4 µg per mouse) was subcutaneously injected into the left axilla of mice. Tumors from each treatment group were collected for flow cytometry and cytokine assays. In the flow cytometry study, M2‐TAMs and M1‐TAMs were identified as CD11b^+^F4/80^+^CD206^+^ cells and CD11b^+^F4/80^+^CD86^+^ cells, respectively. As presented in **Figure** **5**A,D, DOX and Lip displayed very weak effects on the depletion of M2‐TAMs. By contrast, FA‐Lip and PEG‐FA‐Lip considerably reduced the number of M2‐TAMs in tumors. After PEG‐FA‐Lip treatment, the number of M2‐TAMs was 61.6% lower than that in the saline group. In addition, FA‐Lip and PEG‐FA‐Lip had higher numbers of M1‐TAMs which was proved to facilitate the induction of antitumor immunity (Figure 5B,E). The increased accumulation of M1‐TAMs might be explained as follow: on the one hand, PEG‐FA‐Lip had higher toxicity to M2‐TAMs due to the FA modification. When reaching to tumor sites, PEG‐FA‐Lip responded to MMP2 and then was preferentially phagocytized by M2‐TAMs rather than by M1‐TAMs, thus contributing a lower toxicity to M1‐TAMs. On the other hand, the elimination of abundant M2‐TAMs would downregulate the secretion of immunosuppressive cytokines, which could be beneficial for TAMs polarizing to the activated M1 phenotype.^33^ Those results were consistent with that of the MTT assay in vitro (Figure S3, Supporting Information), which demonstrated that PEG‐FA‐Lip could efficiently eliminate M2‐TAMs in tumor sites. Furthermore, the number of M2‐TAMs in CpG/PEG‐FA‐Lip treated mice was closed to that of PEG‐FA‐Lip group, demonstrating CpG had no significant effect on depletion of M2‐TAMs by PEG‐FA‐Lip (Figure 5A,D).

M2‐TAMs secrete large amounts of immunosuppressive cytokines and increase the population of Treg cells to disable effector T cells in tumors.[[qv: 33b,34]] The depletion of M2‐TAMs is believed to remold the immunosuppressive tumor microenvironment. To test this, we first investigated the abundance of Treg cells in tumors. Treg cells were identified as CD4^+^CD25^+^Foxp3^+^ cells (Figure 5C,F). Results revealed that the amount of Treg cells in PEG‐FA‐Lip treated tumor was significantly decreased, which was just 26.5% of that in saline group. In addition, the number of Treg cells in CpG/PEG‐FA‐Lip treated mice was closed to that of PEG‐FA‐Lip group. Next, we evaluated immunosuppressive cytokines secretion including TGF‐β1 and IL‐10 in tumors.[[qv: 16a]] PEG‐FA‐Lip displayed the extremely low levels of TGF‐β1 and IL‐10 expression in tumors, which was 2.6‐fold and 3.5‐fold lower than that of the saline group (Figure 5G,H). CpG/PEG‐FA‐Lip treated mice had similar levels of TGF‐β1 and IL‐10 expression in tumors compared with PEG‐FA‐Lip treated group (Figure 5G,H). These results demonstrated that CpG had negligible effect on PEG‐FA‐Lip reducing Treg cell amount and immunosuppressive cytokine secretion. The combination of CpG with PEG‐FA‐Lip could efficiently remold the immunosuppressive tumor microenvironment.

### 2.5 Antitumor Immunity of PEG‐FA‐Lip Combining with CpG

**Figure 6:**
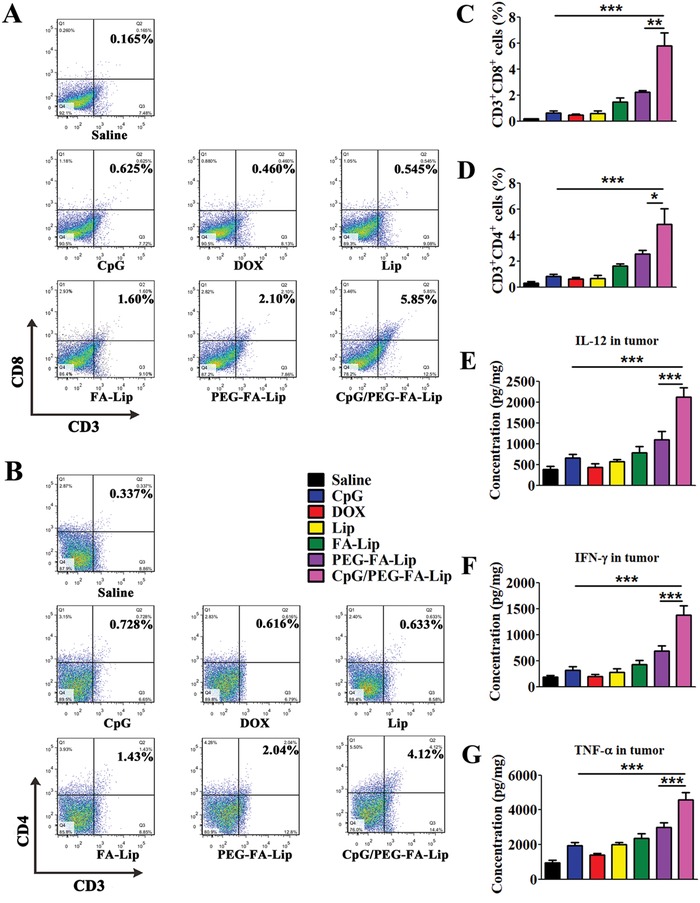
Antitumor immunity of combining CpG with PEG‐FA‐Lip. A,B) Representative flow cytometry profiles of CD3^+^CD8^+^ cells (CD8+ T cells, A) and CD3^+^CD4^+^ cells (CD4+ T cells, B) in 4T1 tumors after the indicated treatment. C,D) Relative abundance of CD8+ T cells (C) and CD4+ T cells (D) in 4T1 tumors after the indicated treatment. E–G) Immunostimulatory cytokines secretion. ELISA results of IL‐12p70 (E), IFN‐γ (F), and TNF‐α (G) secretion 4T1 tumor tissues after the indicated treatment. * *P* < 0.05, ** *P* < 0.01, *** *P* < 0.001. Data represent mean ± SD (*n* = 5).

The efficient effector T cells activation and decreased tumor immunosuppression could contribute to effector T cells arousing robust antitumor immunity within tumor.^35^ To confirm this, we investigated the abundance of effector T cells and the secretion of immune cytokines in tumor tissues. Quantitative flow cytometry results showed that combining CpG with PEG‐FA‐Lip treatment led to highest levels of CD4+ T cells and CD8+ T cells in tumors (**Figure** **6**A,B). In addition, the percentages of CD4+ T cells and CD8+ T cells in CpG/PEG‐FA‐Lip group were 2.79‐fold and 2.02‐fold higher, respectively, than those of PEG‐FA‐Lip group (Figure 6C,D). Furthermore, the secretion of cytokines including TNF‐α, IL‐12p70, and IFN‐γ in tumors could cause tumor regression.^36^ Of note, CpG/PEG‐FA‐Lip showed the highest of those cytokines levels among all treated groups (Figure 6E–G). The results clarified that combining CpG with PEG‐FA‐Lip induced a strong antitumor immunity.

### 2.6 Antitumor Effect

**Figure 7:**
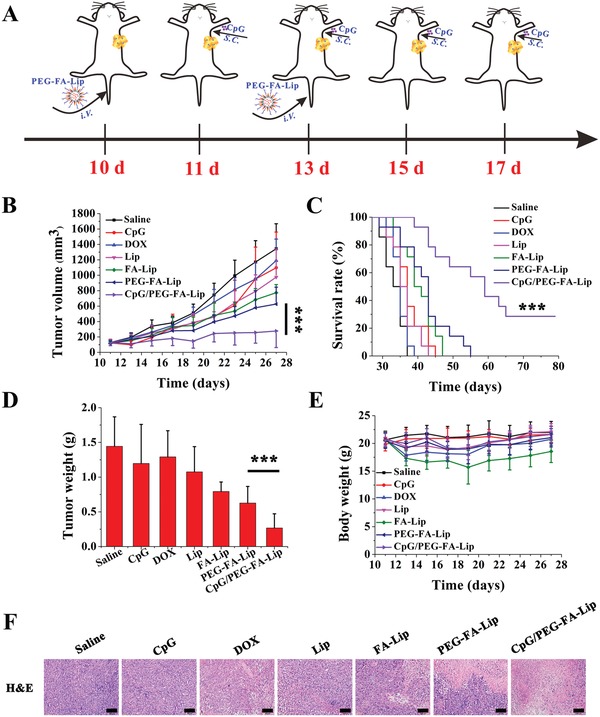
In vivo antitumor efficacy of PEG‐FA‐Lip in combination with CpG. A) Schematic illustration of PEG‐FA‐Lip and CpG combination treatment. B) Tumor growth curves of 4T1 bearing‐mice after being intravenously injected with DOX, Lip, FA‐Lip, and PEG‐FA‐Lip at day 10 and 13, followed by subcutaneous administration of CpG at day 11, 13, 15 and 17 (DOX dose, 5 mg kg^−1^; CpG dose, 4 µg per mouse). Data represent mean ± SD (*n* = 13). C) Kaplan–Meier survival curves of different formulations‐treated 4T1 tumor‐bearing mice. Data represent mean ± SD (*n* = 13). D) Weights of excised tumors at the endpoint of the experiment. Data represent mean ± SD (*n* = 13). *** *P* < 0.001. E) Body weights of 4T1 bearing‐mice from different groups. Data represent mean ± SD (*n* = 13). F) H&E staining of the 4T1 tumor tissues collected at the endpoint of the experiment from different groups of mice (*n* = 5). Scale bar = 100 µm.

Next, the antitumor effect of combining CpG with PEG‐FA‐Lip was confirmed in a 4T1 tumor‐bearing mice model (**Figure** **7**A). Saline and various DOX loaded liposomes were intravenously injected into mice (dose of 5 mg kg^−1^ DOX), respectively. CpG (4 µg per mouse) was subcutaneously injected into the left axilla of mice. Single use of DOX or CpG resulted in limited inhibition of tumor growth, and the tumor volumes were comparable to that of saline group (Figure 7B). Entrapping DOX into Lip and FA‐Lip slightly increased the antitumor efficacy, as indicated by 27.4% and 41.7% reduction of tumor volume, respectively, compared with the saline group. PEG‐FA‐Lip had better inhibitory effect against tumor growth than FA‐Lip. Of note, CpG/PEG‐FA‐Lip treated mice showed the highest inhibition rate of tumor growth, with 84.1% reduction in tumor volume compared with saline group. Furthermore, 30.1% of mice treated with CpG/PEG‐FA‐Lip achieved the goal of tumor eradication (Figure 7B). Besides, we recorded the weights of tumor tissues from all treated groups (Figure 7D), which further confirmed that CpG/PEG‐FA‐Lip was the most effective in suppressing tumor growth. Furthermore, mice treated with CpG/PEG‐FA‐Lip displayed the longest whole survival (Figure 7C). The median survival of female BALB/c mice bearing 4T1 tumors treated with CpG/PEG‐FA‐Lip (57.0 days) was significantly longer than those of mice treated with saline (31.0 days, *P* < 0.001), DOX (33.0 days, *P* < 0.001), Lip (33 days, *P* < 0.001), FA‐Lip (37.0 days, *P* < 0.001), and PEG‐FA‐Lip (41.0 days, *P* < 0.001) (Table S2, Supporting Information). Usually, CpG monotherapy shows efficient antitumor effect against tumors with a small size (<20 mm^3^) but limited efficacy on large solid tumors as advanced tumors with mature microenvironment were more immunosuppressive.[[qv: 4b,37]] PEG‐FA‐Lip remolded immunosuppressive tumor microenvironment by the significant depletion of M2‐TAMs (Figure 5A). Furthermore, with FA modification, PEG‐FA‐Lip targetedly delivered DOX into tumor cells and M2‐TAMs rather than immunocytes, which could protect activated effector T cells from killing by DOX in tumor. Therefore, CpG/PEG‐FA‐Lip displayed impressive efficacy against breast cancers with tumor size over 100 mm^3^. It was also reported that CpG and chemotherapeutic drugs were coloaded in drug carrier and targetedly delivered into tumor.^38^ In such strategy, DCs in tumor would be easily killed by chemotherapeutic drugs for DCs were very sensitive to chemotherapeutic agents as reported in our previous study.^28^ In addition, CpG plays effect via acting on immune cells, and immune organs are the main places to trigger immune response.[[qv: 30a,39]] Thus delivering CpG to tumor sites rather than immune organs is hard to ensure an efficient immune response. By comparison, in our treatment strategy, CpG was locally administered to TDLNs site, which could ensure the DC maturation in TDLNs and was advantageous to arouse systematic immune response. Meanwhile, the chemotherapeutic drugs were selectively delivered into tumor cells and M2‐TAMs, consequently protecting the immune cells in tumor sites from the toxicity of antitumor drugs.

To further confirm the antitumor efficacy of the combined therapy, tumors harvested 2 days after the last treatment were processed for hematoxylin and eosin (H&E) staining. Results of H&E assay revealed that saline group had little apoptosis or necrosis with intact nuclear morphology (Figure 7F). The tumor tissue section samples from Lip and FA‐Lip treated groups exhibited considerable apoptosis or necrosis compared with DOX and CpG. Both PEG‐FA‐Lip and CpG/PEG‐FA‐Lip caused significantly higher levels of apoptosis or necrosis. Remarkably, CpG/PEG‐FA‐Lip group displayed the largest area of nuclei deficient and cell apoptosis (Figure 7F).

In addition, we also evaluated the in vivo safety of CpG/PEG‐FA‐Lip. Major organs (heart, liver, spleen, lung, and kidney) and blood were also collected 2 days after the last treatment. The results revealed that FA‐Lip caused obvious toxicity to spleen and liver. PEG‐FA‐Lip remarkably reduced the unexpected toxicity of DOX to heart, liver, spleen, and bone marrow (Figures S8 and S9, Supporting Information). In addition, CpG/PEG‐FA‐Lip also showed significantly decreased the unexpected toxicity of DOX. These results demonstrated that the combination of PEG‐FA‐Lip with CpG had good in vivo safety.

### 2.7 Distant Tumor Growth Inhibition

**Figure 8:**
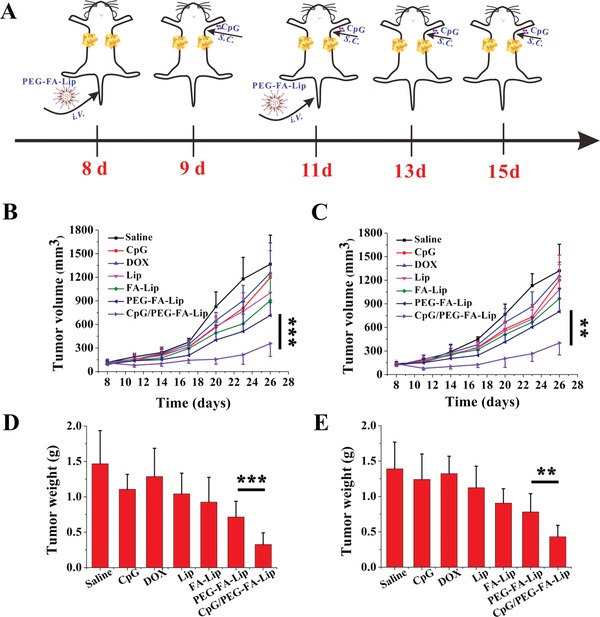
Distant tumor growth inhibition effect of PEG‐FA‐Lip in combination with CpG. A) Schematic illustration of PEG‐FA‐Lip and CpG combination treatment. Mice with 4T1 tumors on both sides were intravenously injected with DOX, Lip, FA‐Lip, and PEG‐FA‐Lip at day 8 and 11, followed by subcutaneous administration of CpG into left axilla at day 9, 11, 13, and 15 (DOX dose, 5 mg kg^−1^; CpG dose, 4 µg per mouse). Tumors on the left side were designated as “primary tumors” and those on the right side were designated as “distant tumors.” B,C) Growth curves for primary tumors (B) and distant tumors (C) on mice after the indicated treatment. D,E) Weights of primary tumors (D) and distant tumors (E). ** *P* < 0.01, *** *P* < 0.001. Data represent mean ± SD (*n* = 8).

Tumor metastasis is the main killer in breast cancers and accounts for the extremely low survival rate.^40^ M2‐TAMs were reported to significantly increase the incidence of tumor metastasis by vascular endothelial growth factor (VEGF) expression.^17^, ^41^ With the exceptional ability of M2‐TAMs depletion (Figure 5A; Figure S10, Supporting Information) and effector T cell activation (Figure 4), the combination of CpG with PEG‐FA‐Lip had extraordinary inhibition effect against tumor cells circulating in the body and could effectively inhibit lung metastasis of breast cancers (Figure S11, Supporting Information). However, we should pay more attention to the treatment of the already metastasized solid tumors in which there is a mature microenvironment. Therefore, we developed a bilateral breast tumor model by subcutaneously injecting 4T1 cells into both the left and right flank of mice. The left and right tumors were designated as the primary and distant tumors, respectively. As shown in **Figure** **8**B,C, PEG‐FA‐Lip together with local CpG therapy efficiently controlled the growth of both primary and distant tumor (initial tumor volume > 100 mm^3^). In particular, CpG/PEG‐FA‐Lip dramatically reduced the distant tumor volume by 69.6% compared with the saline group, which was 3.0‐fold and 2.0‐fold times that of CpG and PEG‐FA‐Lip group, respectively. The same trend also observed in the results of the tumor mass study (Figure 8D,E). CpG/PEG‐FA‐Lip treated group had the smallest primary and distant tumor masses among all groups. At the end of the study, distant tumors were collected for immunohistochemical analysis, and the immunohistochemical images revealed that CpG/PEG‐FA‐Lip significantly increased the accumulation of CD8^+^ T cells and CD4^+^ T cells in tumor tissues compared with other treated groups (Figure S12, Supporting Information). These results implied that CpG/PEG‐FA‐Lip triggered the systemic antitumor immune response and had impressive therapeutic effects on metastasized tumors. Tumor metastasis is the main death reason for most cancer patients.[[qv: 40a,42]] Recently, many researches[[qv: 3b,43]] have proved that photothermal therapy combined with immune adjuvant could also conduct the antitumor immune response to inhibit tumor metastasis. However, photothermal therapy could not overcome the tumor microenvironment of distant tumor, thus the antitumor immune response triggered by photothermal therapy usually showed the effective antitumor effect on distant tumor with tumor volume of less than 20 mm^3^.[[qv: 3b,43]] Nevertheless, our treatment strategy could effectively control the distant tumor with tumor volume of more than 100 mm^3^ for our drug delivery system could reprogram immunosuppressive tumor microenvironment not only in primary tumor but also in distant tumor.

## 3 Conclusions

We developed a combination therapeutic strategy that could arouse robust antitumor immunity against solid tumors by efficiently activating effector T cells and reversing tumor immunosuppression. We proved that PEG‐FA‐Lip could target both 4T1 tumor cells and M2‐TAMs, thus contributing to extensive “tumor vaccines” and tumor immunosuppression reduction. The combination of CpG immune adjuvant remarkably facilitated PEG‐FA‐Lip induced “tumor vaccines” to trigger effector T cell activation. We also confirmed the impressive therapeutic efficacy of PEG‐FA‐Lip combining with CpG in the treatment of systemic and metastatic breast cancers. Such combined strategy achieved synergistic therapeutic efficacy and had great potential in eradicating primary tumors, preventing tumor metastasis and inhibiting distant tumors.

## 4 Experimental Section

*Materials*: 1,2‐distearoyl‐sn‐glycero‐3‐phosphoethanolamine‐*N*‐[methoxyn(polyethyleneglycol)‐1000] (mPEG_1000_‐DSPE), 1,2‐distearoyl‐sn‐glycero‐3‐phosphoethanolamine‐*N*‐[maleimide (polyethylene glycol)‐1000] (MAL‐PEG_1000_‐DSPE), and Lipoid E80 (purified ovolecithin) were purchased from Lipoid Co., Ltd. (Ludwigshafen, Germany). Folate‐PEG_1000_‐DSPE was custom‐synthesized by Pong Sheng Biological Ltd. (Shanghai, China) and mPEG_2000_‐MMP2 cleavable peptide‐Cys was synthesized by GL Biochem (Shanghai) Ltd. (Shanghai, China). CpG‐ODN 1826 (5′‐tccatgacgttcctgacgtt‐3′; total backbone phosphorothioated) was gained from Shanghai Sangon Biological Engineering Technology & Services (Shanghai, China). Doxorubicin hydrochloride was obtained from Huafenglianbo Technology Co., Ltd. (Beijing, China). Cholesterol and lipopolysaccharides (LPS) were obtained from Sigma‐Aldrich Co., LLC. (St. Louis, USA). DiD (1,1′‐dioctadecyl‐3,3,3′,3′‐tetramethyl indodicarbocyanine, 4‐chlorobenzenesulfonate salt) was purchased from Biotium (Hayward, USA). Human active MMP2 protein (MW 66 000 Da) and Interleukin 4 (IL‐4) was obtained from Pepro Tech (Rocky Hill, USA). Antibodies against cell surface markers for flow cytometry assay were purchased from Affymetrix eBioscience Co., Ltd. (San Diego, USA).

*Cells and Mice*: 4T1 mouse breast cancer cells, Raw 264.7 cells and DC 2.4 cells were purchased from Chinese Academy of Science Cell Bank for Type Culture Collection (Shanghai, China). All cells were cultured in RPMI‐1640 medium containing 10% fetal bovine serum and 100 U mL^−1^ penicillin–streptomycin under 5% CO_2_ at 37 °C. Female BALB/c mice (6–8 weeks old) were provided by Chengdu Dossy Biological Technology Co., Ltd. (Chengdu, China). All animal studies were conducted the according to the requirements of the national act on the use of experimental animals (China) and in compliance with guidelines evaluated and approved by the Animal Ethics Committee of Sichuan University.

*Preparation and Characterization of Lip, FA‐Lip, and PEG‐FA‐Lip*: Liposomes were prepared by the thin‐film hydration method.^25^ In brief, E80, cholesterol, mPEG_1000_‐DSPE, and MAL‐PEG_2000_‐DSPE (molar ratio, 40:25:2:3) were dissolved in chloroform, and the organic solvent was removed by rotary evaporation at 37 °C to gain the thin film. The lipid thin was hydrated with 200 × 10^−3^ m ammonium sulfate at 37 °C followed by sonicating for 6 min to form maleimide functionalized liposome (MAL‐Lip). DOX was entrapped into blank liposomes by the ammonium sulfate gradient method.[[qv: 19a]] The unencapsulated DOX was removed by elution through a Sephadex G‐75 column. Lip were prepared as described earlier using mPEG_1000_‐DSPE instead of MAL‐PEG_1000_‐DSPE. FA‐Lip was prepared as indicated above using E80, cholesterol, mPEG_1000_‐DSPE, and Folate‐PEG_1000_‐DSPE (molar ratio, 40:25:3:2). PEG‐FA‐Lip was prepared by maleimide‐thiol coupling reaction at room temperature for 24 h. Unconjugated mPEG_2000_‐MMP2 cleavable peptide was removed by elution through a Sephadex G‐75 column.

The particle size and zeta potential of Lip, FA‐Lip, and PEG‐FA‐Lip were determined by dynamic light scattering using Zetasizer Nano ZS90 instrument (Malvern Panalytical, Malvern, UK). The encapsulation efficiency (EE %) was determined according to the previous report.[[qv: 19a]] The morphology of liposomes was performed by TEM (H‐600, Hitachi, Japan).

*Cellular Uptake*: RAW 264.7 cells were treated with 50 ng mL^−1^ LPS or 20 ng mL^−1^ IL‐4 for 48 h to stimulate M1 or M2 polarization in macrophages. 4T1 cells and polarized RAW264.7 cells were seeded in 12‐well plates at a density of 1 × 10^5^ cells per well and allowed to grow for 24 h. Then, cells were treated with Lip, FA‐Lip, and PEG‐FA‐Lip with or without incubation with 5 µg mL^−1^ of MMP2 in 1 mL per well of the serum‐free medium. For competitive inhibition assay, cells were preincubated with 400 µg mL^−1^ of FA solution for 1 h. After 1 h incubation, cells were trypsinized, then centrifuged at 2000 rpm for 3 min, collected and suspended in PBS. The intensity of drug fluorescence was measured by flow cytometer (BD FACSCelesta, USA).

For the qualitative analysis of cellular uptake, cells seeded in glass‐bottomed dishes at a density of 1 × 10^5^ cells mL^−1^ were treated as the aforementioned protocols. After being incubated with various liposomes for 1 h, cells were fixed with 4% polyoxymethylene for 15 min, and stained with DAPI for 5 min at a darkness environment. After that, cells were washed thrice with PBS. The fluorescent images were acquired using a laser scanning confocal microscope (Olympus Fluoview FV 1000, USA).

*In Vivo Dual‐Targeting Efficacy of PEG‐FA‐Lip*: To establish the model of breast tumor‐bearing mice, 5 × 10^5^ 4T1 cells suspended in 100 µL PBS were subcutaneously injected into the left flank of female BALB/c mice (6–8 weeks old). After tumor cells implantation for 21 days, mice were intravenously administrated with DiD‐loaded Lip, FA‐Lip, and PEG‐FA‐Lip via tail vein. At 2, 8, and 24 h after administration, the fluorescence biodistribution of DiD in tumor was analyzed. At the end of this experiment, mice were sacrificed, and blood, organs (heat, liver, spleen, lung, and kidney) and tumors were collected for ex vivo imaging of DiD fluorescence by Caliper IVIS Lumina III (Perkin elmer, USA).

Breast tumor‐bearing mice were treated with DiD‐loaded Lip, FA‐Lip, and PEG‐FA‐Lip via intravenous injection. After 24 h, mice were sacrificed and tumors were excised. Tumor frozen sections of 10 µm thickness were stained with mouse anti‐F4/80 and anti‐CD206 antibodies for M2‐TAMs. DAPI was used for the nuclear stain. The fluorescent images were taken with a laser scanning confocal microscope.

For pharmacokinetic studies, male SD rats were intravenously injected 3 mg kg^−1^ of DOX solution, FA‐Lip, and PEG‐FA‐Lip. Blood was withdrawn at preset time intervals and centrifuged at 5000 rpm for 5 min. The gained plasma was stored at −80 °C for further analysis.

*Immunogenic Cell Death Analysis*: For the apoptosis and HMGB1 release studies, 4T1 cells were seed in 12‐well plates. Then, 400 ng mL^−1^ DOX equivalents of Lip, FA‐Lip, and PEG‐FA‐Lip with/without preincubation with 5 µg mL^−1^ of MMP2 in 1 mL per well of the serum‐free medium were added, respectively. After incubation for 24 h, the 4T1 tumor cell apoptosis and HMGB1 release were determined with an Annexvin V‐FITC Apoptosis/Propidium Iodide Detection kit (Keygen, Nanjing, China) and ELISA kit (IBL International, Hamburg, Germany), respectively, according to the manufacturer's instructions.

For the CRT exposure assay, 4T1 cells were seeded in glass‐bottomed dishes treated as described for the apoptosis study. After incubation for 24 h, cells were fixed with 4% polyoxymethylene for 15 min, followed staining with Alexa Fluor 488‐CRT antibody for 1 h and DAPI for 5 min in a dark environment. Finally, these prepared cell samples were observed with confocal laser scanning microscopy (Olympus Fluoview FV 1000, USA).

For the DC phagocytosis study, 4T1 cells were seeded in 12‐well plates and treated with 400 ng mL^−1^ DOX equivalents of Lip, FA‐Lip, and PEG‐FA‐Lip for 24 h. Then 4T1 cells were collected and labeled with Vybrant DiO cell labeling solution (Invitrogen). The DC 2.4 cells were dye labeled with Vybrant DiI cell labeling solution (Invitrogen) before being cocultured with DiO labeled 4T1 tumor cells. DC2.4 cells seeded in glass‐bottomed dishes were fed with 4T1 tumor cells at a DC/tumor cell ratio of 1:5. After that, cells were fixed with 4% polyoxymethylene for 15 min, and washed thrice with PBS. The phagocytosis of DOX loaded liposomes treated 4T1 cells by DC 2.4 cells was observed by confocal laser scanning microscopy analysis.

For the evaluation of ICD in vivo, female BALB/c mice bearing 4T1 breast cancers were treated with 2 doses of various DOX formulations at day 10 and 13. Two days after last treatment, the tumor tissues were collected for TUNEL, CRT, and HMGB1 staining.

*CpG Distribution in TDLNs*: Female BALB/c mice bearing 4T1 breast cancers were established and were divided into two groups. After tumor cells implantations for 11 days, the CpG/PEG‐FA‐Lip group were intravenously administrated PEG‐FA‐Lip via tail vein. At day 12, CpG‐FITC (4 µg per mouse) was subcutaneously injected into the left axilla of mice. The TDLNs at the injection sites were harvested at 1, 8, and 24 h after immunization. The fluorescence biodistribution of CpG‐FITC in tumor was analyzed.

*Dendritic Cell Maturation and Effector T Cells Activation in TDLNs*: Female BALB/c mice bearing 4T1 breast cancers were established as described before. At day 10 and 13, saline and 5 mg kg^−1^ DOX equivalents of DOX, Lip, FA‐Lip, and PEG‐FA‐Lip were intravenously injected into mice. At day 11, 13, 15, and 17, CpG (4 µg per mouse) was subcutaneously injected into the left axilla of mice. Two days after last treatment, TDLNs located at the left axilla were collected. To prepare single cell suspension, TDLNs were weighted, excised, and forced through a 70 µm cell strainer. Then cells were centrifuged, washed, and suspended in PBS. The cell suspensions were costained with FITC‐conjugated antibody against CD11c (to mark dendritic cells), PE/Cy5‐conjugated antibody against CD86 and PE‐conjugated antibody against CD80 for flow cytometry assay. To investigate effecort T cells activation, the single cell suspensions were incubated with antibodies of PE anti‐CD4, FITC anti‐CD8a, and APC anti‐CD3e for flow cytometry assay.

*Analysis of Tumor‐Infiltrating Leukocytes*: 4T1 breast cancer‐bearing mice were treated as indicated for the dendritic cell maturation study. Tumors were harvested 2 days after the last treatment and the single cell supernatant was prepared according to the previous report.^28^ Tumor tissues were dissected, digested with collagenase and forced through a 70 µm cell strainer. The single cell suspensions were incubated with antibodies. Antibody combinations used to distinguish immune cell populations were listed as follows: CD3^+^, CD4^+^ (CD4+ T cells), CD3^+^, CD8^+^ (CD8+ T cells), CD11b^+^, F4/80^+^, CD86^+^ (M1‐TAMs), CD11b^+^, F4/80^+^, CD206^+^ (M2‐TAMs) and CD4^+^, CD25^+^, Foxp3^+^ (Treg cells). Intracellular mouse Foxp3 staining was conducted using the Anti‐Mouse/Rat Foxp3 Staining Set (Affymetrix eBioscience) according to the manufacturer's instructions. All stained cells were suspended in PBS and assayed by a flow cytometer.

*Cytokine Assay*: 4T1 breast tumor‐bearing mice were treated as described earlier. Blood samples and tumors were collected 2 days after the last treatment. Blood samples were centrifuged at 6000 rpm for 10 min at 4 °C to gain serum. TNF‐α, IL‐12, and IFN‐γ in serum were detected using ELISA kits (Dakewe, China).

Tumors were homogenized on ice with RIPA solution containing protease inhibitor cocktail (Sigma‐Aldrich). The cell debris was removed by centrifugation at 12 000 rpm for 5 min at 4 °C. TNF‐α, IL‐12p70, IFN‐γ, TGF‐β1, and IL‐10 levels in the supernatants of tumors were measured using ELISA kits (Dakewe, China) according to the manufacturer's instructions.

*Antitumor Efficacy*: Female BALB/c mice bearing 4T1 breast cancers were established as indicated above, when the tumor volume reached 100 mm^3^ at day 10, mice were randomly divided into seven groups including saline, CpG, DOX, Lip, FA‐Lip, PEG‐FA‐Lip, and CpG/PEG‐FA‐Lip. At day 10 and 13, saline and 5 mg kg^−1^ DOX equivalents of DOX, Lip, FA‐Lip, and PEG‐FA‐Lip were intravenously injected into mice. At day 11, 13, 15, and 17, CpG (4 µg per mouse) was subcutaneously injected into the left axilla of mice. Tumor volume and mouse weight were measured every other day after first administration. The survival of animals was recorded and presented by Kaplan–Meier plots, and analyzed with Log‐Rank test.

*Immunohistochemical and Histopathological Analysis*: Tumors and major organs (heart, liver, spleen, lung, and kidney) harvested 2 days after the last treatment in antitumor efficacy study and distant tumor growth inhibition effect assay were fixed in 4% paraformaldehyde solution. Then the tumor tissues were embedded in paraffin, sectioned, and processed for H&E, VEGF, CD4^+^, and CD8^+^ staining. These prepared sections were observed by a light microscope (Axiovert 40CFL, Germany).

*Pulmonary Metastasis Study*: Female BALB/c mice bearing 4T1 breast cancers were established as described before. At Day At day 10 and 13, saline and 5 mg kg^−1^ DOX equivalents of DOX, Lip, FA‐Lip, and PEG‐FA‐Lip were intravenously injected into mice. At day 11, 13, 15, and 17, CpG (4 µg per mouse) was subcutaneously injected into the left axilla of mice. 4T1 breast cancers‐bearing mice were sacrificed and lungs were collected 3 weeks following the last treatment. Lungs were fixed in 4% paraformaldehyde solution and photographed. The pulmonary metastatic nodules were counted and analyzed. After that, lungs were embedded into paraffin, sectioned, and stained with H&E for the histological evaluation.

*Distant Tumor Growth Inhibition Effect*: Female BALB/c mice were injected subcutaneously with 5 × 10^5^ 4T1 cells into the left flank (primary tumor) and 5 × 10^5^ 4T1 cells into the right flank (distant tumor). At day 8 and 11, saline and 5 mg kg^−1^ DOX equivalents of DOX, Lip, FA‐Lip, and PEG‐FA‐Lip were intravenously injected into mice. At day 9, 11, 13, and 15, CpG (4 µg per mouse) was subcutaneously injected into the left axilla of mice. The volume of secondary tumor was measured every 2 days and the whole survival was recorded and presented by Kaplan–Meier plots.

*Statistical Analysis*: Statistical analysis was performed by the ANOVA. Survival analysis was determined by Kaplan–Meier method and compared by the Log‐Rank test using the SPSS software. All quantitative parameters were presented as mean with SD (standard deviations). *P* value of <0.05, <0.01, and <0.001 are accepted as indicative of significant differences.

## Conflict of Interest

The authors declare no conflict of interest.

## Supporting information

Supplementary
