# Supplementary material for: Tumors and Their Microenvironment Dual‐Targeting Chemotherapy with Local Immune Adjuvant Therapy for Effective Antitumor Immunity against Breast Cancer
Source: Adv Sci (Weinh). 2019 Jan 30;6(6):1801868. doi: 10.1002/advs.201801868 (PMC6425447; doi:10.1002/advs.201801868)
Supplement: Supplementary file 1 — Supplementary [file ADVS-6-1801868-s001.pdf]

Copyright WILEY-VCH Verlag GmbH & Co. KGaA, 69469 Weinheim, Germany, 2019.

ADVANCED SCIENCE Open Access

Supporting Information

for *Adv. Sci.,* DOI: 10.1002/advs.201801868

Tumors and Their Microenvironment Dual-Targeting Chemotherapy with Local Immune Adjuvant Therapy for Effective Antitumor Immunity against Breast Cancer

Caifeng Deng, Quan Zhang, Mengdi Jia, Jin Zhao, Xun Sun, Tao Gong, and Zhirong Zhang*

SUPPORTING INFORMATION

Tumors and Their Microenvironment Dual-Targeting Chemotherapy with Local Immune Adjuvant Therapy for Effective Antitumor Immunity against Breast cancer

Caifeng Deng, Quan Zhang, Mengdi Jia, Jin Zhao, Xun Sun, Tao Gong* and Zhirong Zhang

Caifeng Deng and Quan Zhang contributed equally.

Dr. Caifeng Deng, Mengdi Jia, Jin Zhao, Prof. Xun Sun, Prof. Tao Gong, Prof. Zhirong Zhang

Key Laboratory of Drug-Targeting and Drug Delivery System of the Education Ministry, Sichuan Engineering Laboratory for Plant-Sourced Drug and Sichuan Research Center for Drug Precision Industrial Technology, West China School of Pharmacy, Sichuan University, Chengdu 610064, P. R. China.

E-mail: gongtaoy@126.com

Dr. Quan Zhang

School of Pharmacy, Chengdu Medical College, Chengdu 610083, China

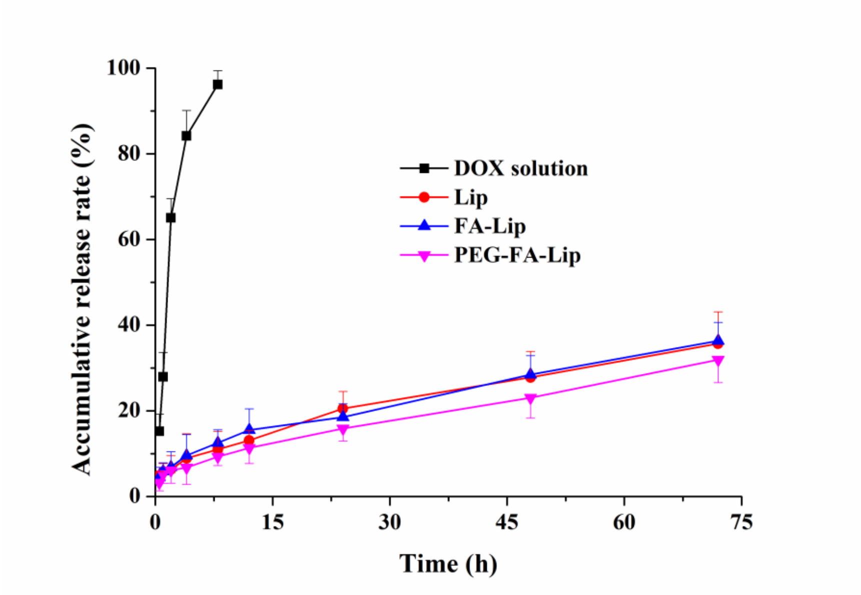

Supplemental Figure 1. DOX release profiles from Lip, FA-Lip and PEG-FA-Lip in saline at 37 °C.

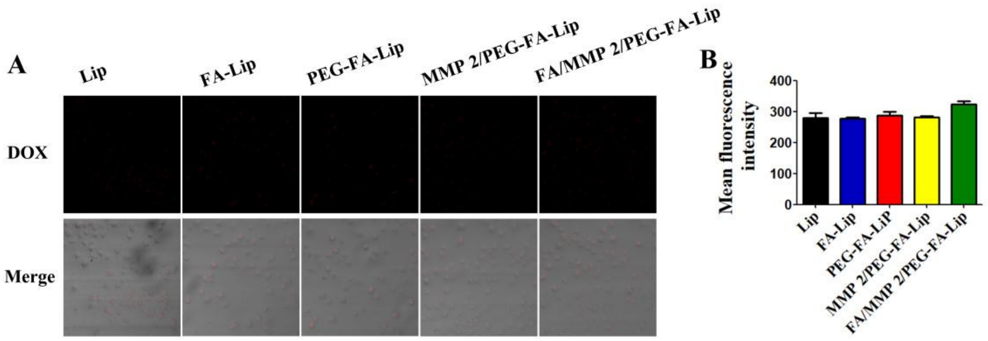

Supplemental Figure 2. (A) Confocal images (200 ×) of cellular uptake on M1 polarized macrophages (RAW 264.7 cells with LPS). (B) Quantitative cellular uptake and of Lip, FA-Lip and PEG-FA-Lip on M1 polarized macrophages after incubation for 1 h at the DOX concentration of 10 μg/mL. Data represent the mean ± SD (n = 3).

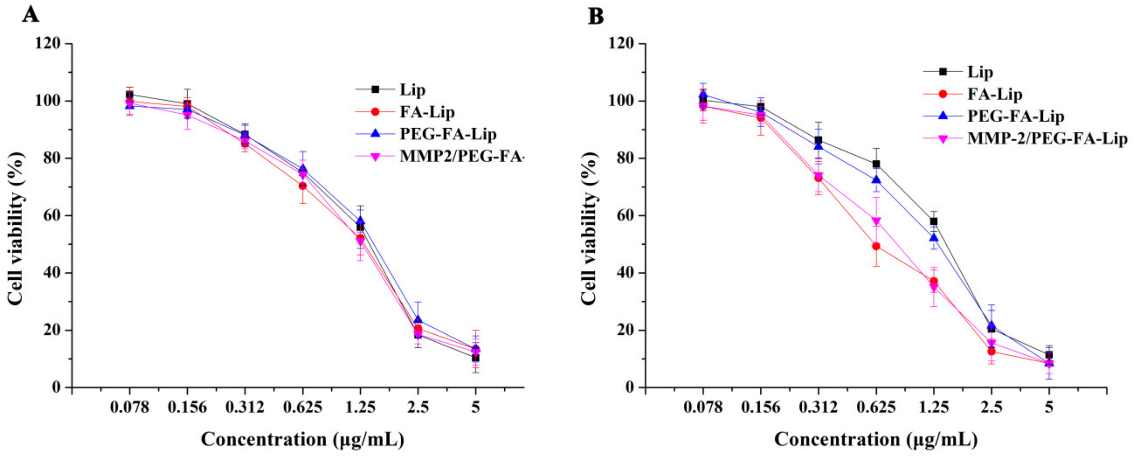

Supplemental Figure 3. MTT assay in vitro. (A, B) Cytotoxicity of various DOX loaded liposomes on M1 polarized macrophages (A) and M2 polarized macrophages (B) after incubation for 24 h. Data represent the mean ± SD (n = 3).

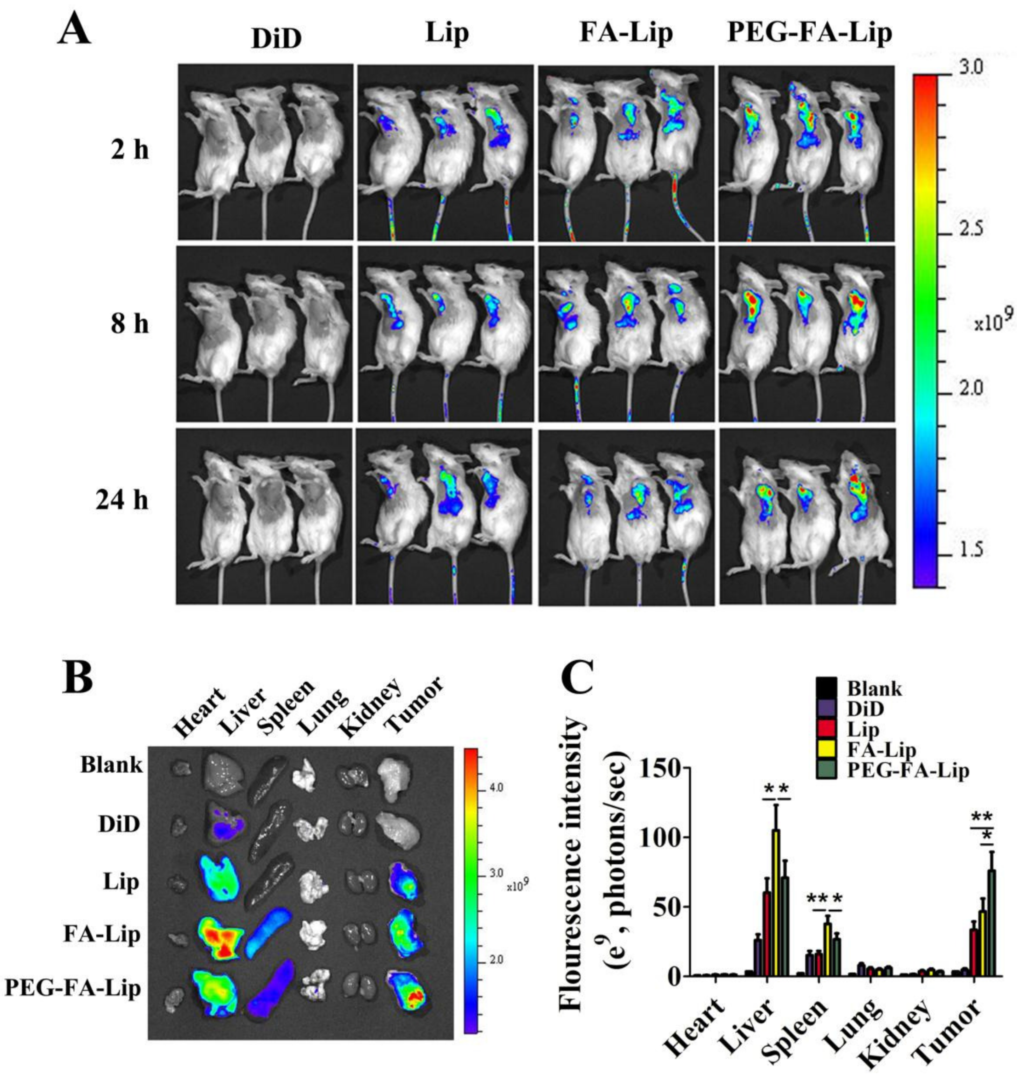

Supplemental Figure 4**.** In vivo dual-targeting efficacy of PEG-FA-Lip. (A) In vivo DiD fluorescence images showing the tumor distribution of Lip, FA-Lip and PEG-FA-Lip in 4T1 tumor-bearing mice at 2, 8 and 24 h post-injection. (B) Ex vivo DiD fluorescence images of major organs and tumors dissected from 4T1 tumor-bearing mice 24 h post injection. (C) The statistical graph of the fluorescence intensity of major organs and tumors based on the semiquantitative analysis of the ex vivo fluorescence images. *P < 0.05, **P < 0.01. Data represent the mean ± SD (n = 3).

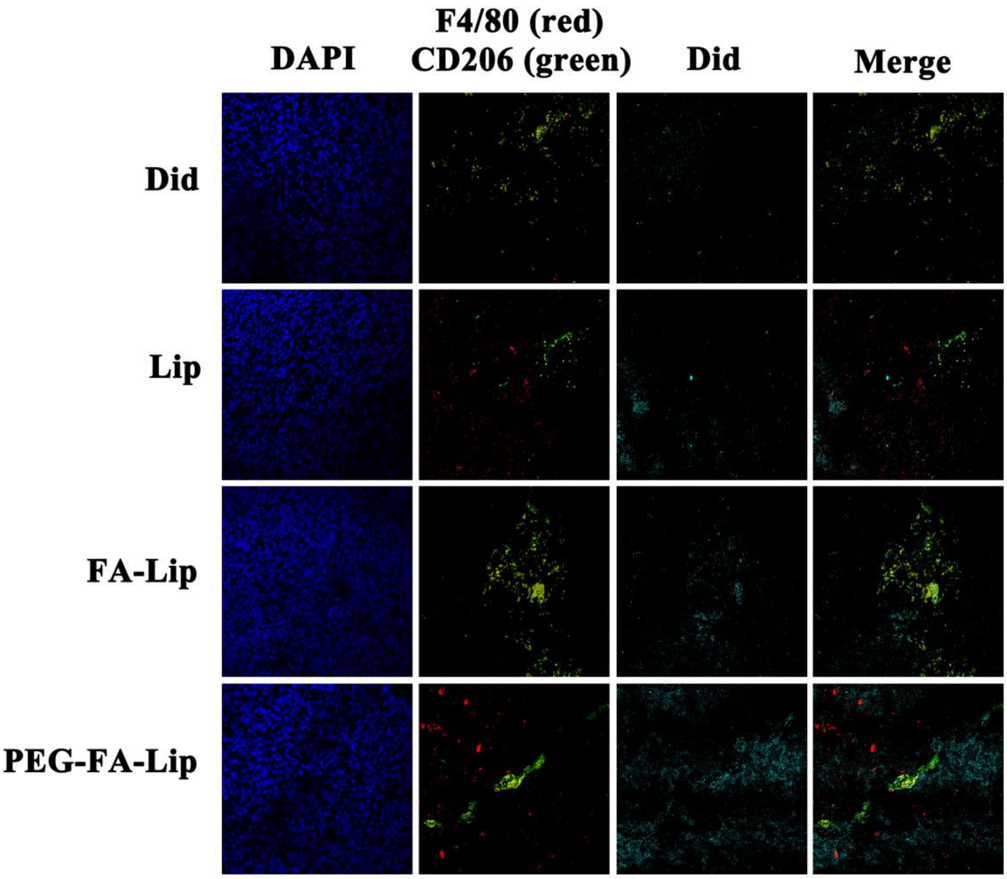

Supplemental Figure 5**.** Confocal images (200 ×) showing the colocalization of different DiD formulations and M2-TAMs. M2-TAMs were determined by double immunofluorescence analysis of CD206 (green fluorescence) and the macrophage maker F4/80 (red fluorescence) (n = 3).

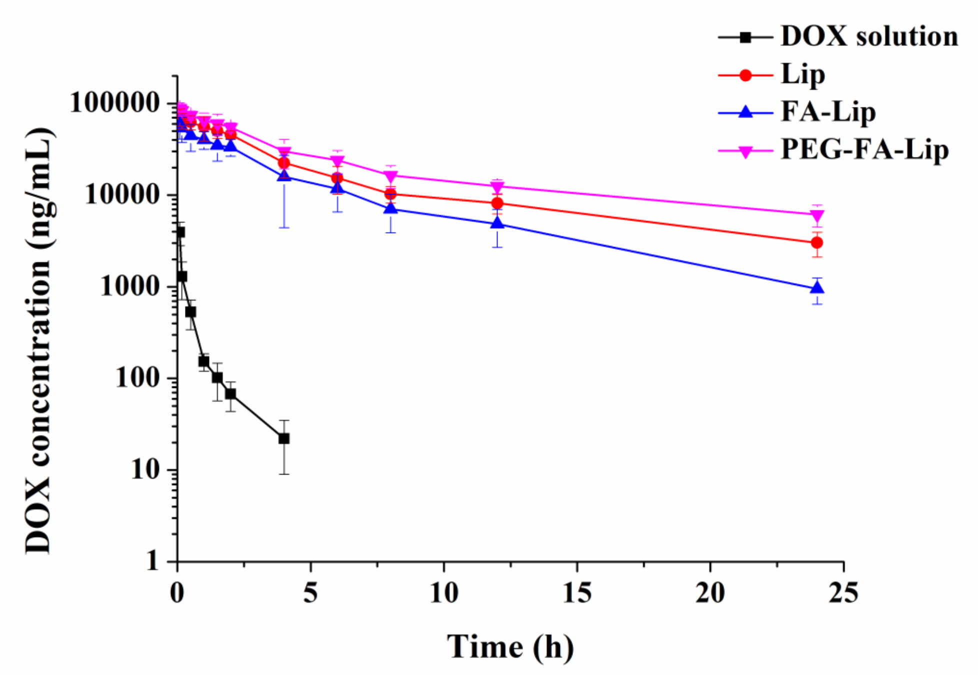

Supplemental Figure 6. Pharmacokinetics behaviors of Lip, FA-Lip and PEG-FA-Lip in rats after intravenous injection of different DOX formulations at an equivalent dose of 3 mg/kg of DOX.

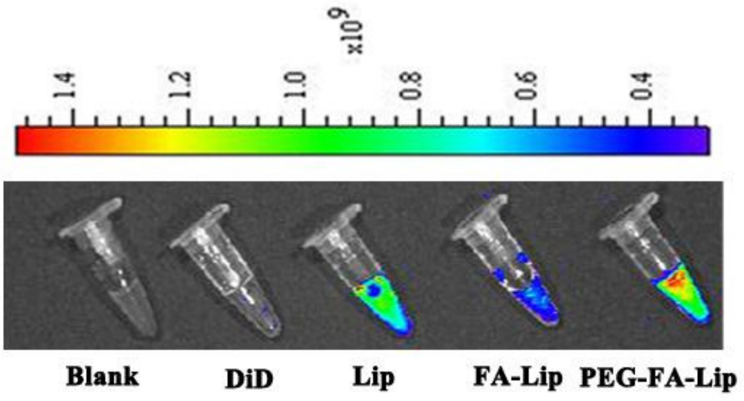

Supplemental Figure 7. Ex vivo DiD fluorescence images of blood collected from 4T1 tumor-bearing mice 24 h after the indicated treatment.

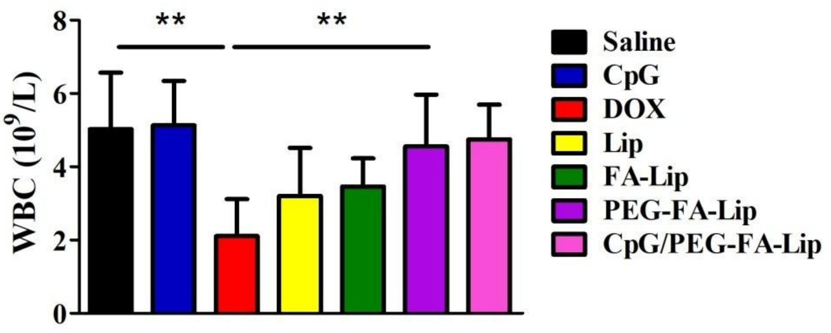

Supplemental Figure 8. Bone marrow toxicity assessment in vivo. White blood cells (WBC) counts of 4T1 tumor bearing-mice were detected 2 days after last treatment in antitumor efficacy study (n = 5). **P < 0.01.

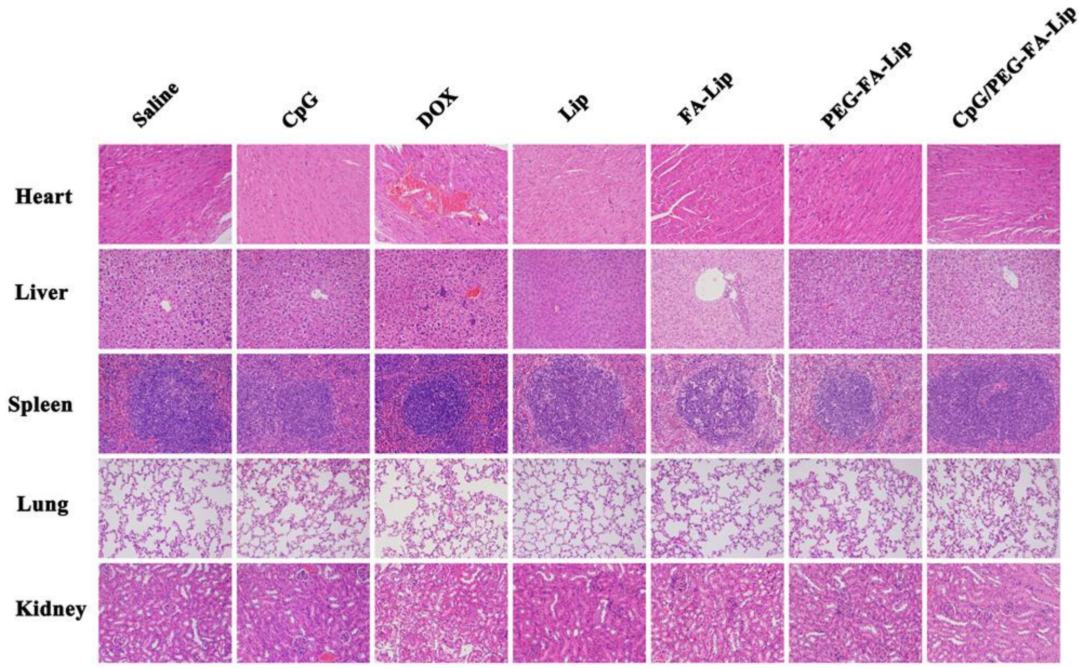

Supplemental Figure 9. Hematoxylin and eosin (H&E) staining of the major organs (heart, liver, spleen, lung and kidney) collected from 4T1 tumor bearing-mice were processed 2 days after last treatment in antitumor efficacy study (magnification 200 ×) (n = 5).

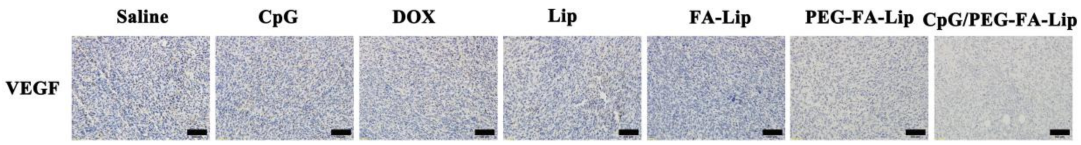

Supplemental Figure 10. VEGF staining of the 4T1 tumor tissues collected at the endpoint of the experiment from different groups of mice in pulmonary metastasis inhibition study (n = 5). Scale bar = 100 μm.

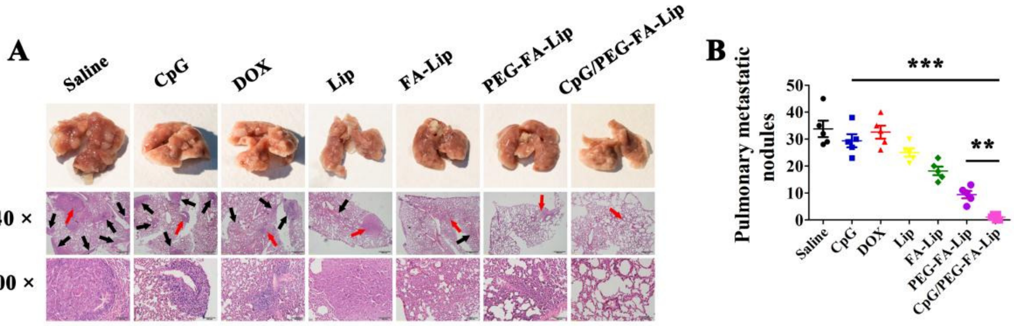

Supplemental Figure 11. Pulmonary metastasis inhibition effect of PEG-FA-Lip in combination with CpG. (A) Representative lung metastatic nodule specimens and H&E staining of lung metastases from mice receiving the indicated treatment. Black arrows in the middle panels point to metastatic 4T1 tumors in lung. Red arrows pointed regions are shown at higher magnification in the lower panels. (B) The average numbers of the pulmonary metastatic nodules in 4T1 tumor-bearing mice at the end of treatment. **P < 0.01, ***P < 0.001. Data represent mean ± SD (n = 5).

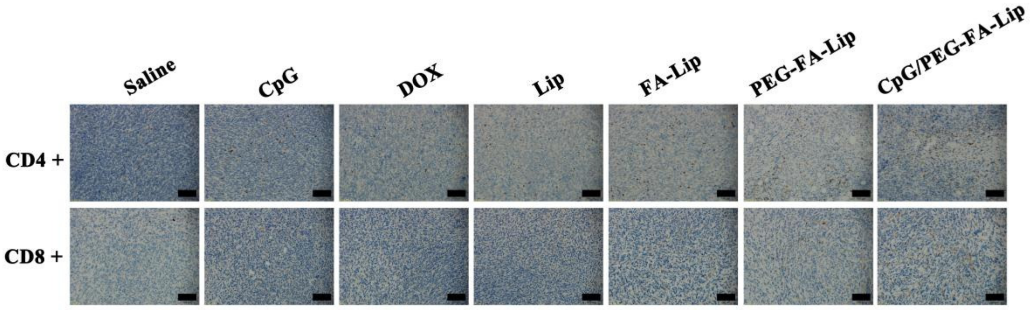

Supplemental Figure 12. $CD8^+$ T cells and $CD4^+$ T cells staining of distant 4T1 tumors tissues from different groups of mice in distant tumor growth inhibition study (n = 5). Scale bar = 100 μm.

Supplemental Table 1. Characterization of the various DOX loaded liposomes. Data represent mean ± SD (n = 3).

	Particle size (nm)	PDI	Zeta potential (mV)	EE (%)
Lip	123.4 ± 5.7	0.232 ± 0.037	-2.4 ± 0.5	92.4 ± 2.4
FA-Lip	127.2 ± 4.9	0.206 ± 0.053	-6.7 ± 0.6	91.6 ± 2.8
PEG-FA-Lip	138.5 ± 6.8	0.190 ± 0.041	-9.3 ± 0.8	93.3 ± 3.1

Supplemental Table 2. In vivo antitumor effects of various DOX loaded liposomes on 4T1 tumor bearing-mice (n = 13). *P < 0.05, **P < 0.01, ***P < 0.001.

Groups	Survival time (days)		Compare with					
	Means (SD)	Medians (SD)	Saline	CpG	DOX	Lip	FA-Lip	PEG-FA-Lip
Saline	31.4 (0.8)	31.0 (1.2)	_	_	_	_	_	_
CpG	35.3 (1.1)	35.0 (0.9)	**	_	_	_	_	_
DOX	33.0 (0.6)	33.0 (0.6)	N.S.	*	_	_	_	_
Lip	34.1 (1.0)	33.0 (0.9)	*	N.S.	N.S.	_	_	_
FA-Lip	38.4 (1.2)	37.0 (1.9)	***	N.S.	***	*	_	_
PEG-FA-Lip	40.3 (1.9)	41.0 (0.8)	***	*	***	**	N.S.	_
CpG/PEG-FA-Lip	58.6 (4.3)	57.0 (3.7)	***	***	***	***	***	***
